# The Traditional Chinese Medicine “Hu-Qian-Wan” Attenuates Osteoarthritis-Induced Signs and Symptoms in an Experimental Rat Model of Knee Osteoarthritis

**DOI:** 10.1155/2022/5367494

**Published:** 2022-02-09

**Authors:** Pu-Wei Hou, Shan-Chi Liu, Gregory J. Tsay, Chih-Hsin Tang, Hen-Hong Chang

**Affiliations:** ^1^Department of Chinese Medicine, China Medical University Hospital, Taichung 40447, Taiwan; ^2^Graduate Institute of Chinese Medicine, China Medical University, Taichung 40402, Taiwan; ^3^Department of Medical Education and Research, China Medical University Beigang Hospital, Yunlin County 65152, Douliu, Taiwan; ^4^School of Medicine, China Medical University, Taichung 40402, Taiwan; ^5^Division of Immunology and Rheumatology, Department of Internal Medicine, China Medical University Hospital, Taichung 40447, Taiwan; ^6^Chinese Medicine Research Center, China Medical University, Taichung 40402, Taiwan; ^7^Department of Biotechnology, College of Health Science, Asia University, Taichung 41354, Taiwan; ^8^Graduate Institute of Integrated Medicine, College of Chinese Medicine, China Medical University, Taichung 40402, Taiwan

## Abstract

**Background:**

Knee osteoarthritis (KOA) is a chronic, low-grade inflammatory disease that affects knee joints and causes functional disability in the elderly. KOA is typically treated with oral NSAIDs, which are commonly associated with gastrointestinal side effects or cardiovascular complications. Traditional Chinese medicine (TCM) is widely used by patients with KOA in Taiwan; the Hu-Qian-Wan (HQW) formula is typically prescribed. We investigated the therapeutic role of a modified version of the HQW decoction in Sprague-Dawley rats with KOA induced by anterior cruciate ligament transection (ACLT) of the right knee.

**Materials and Methods:**

Thirty rats were randomly assigned to five groups (six animals each): arthrotomy alone (sham surgery, controls), ACLT only, ACLT + low-dose (1,000 mg/kg) HQW, ACLT + high-dose (3,000 mg/kg) HQW, and ACLT + celecoxib (30 mg/kg). All study groups underwent weight-bearing behavioral testing, micro-computed tomography (CT), and histological examinations of the knee joint cartilage, as well as immunohistochemical analyses of levels of interleukin (IL) 1*β* and tumor necrosis factor (TNF) *α* expression in articular cartilage.

**Results:**

At 6 weeks, compared with ACLT group only, ACLT rats administered high-dose HQW or celecoxib exhibited the fewest weight-bearing deficits, the greatest improvements from baseline in articular cartilage architecture, and the lowest amounts of TNF-*α* and IL-1*β* staining in cartilage and synovial sections (all values were significant compared with the ACLT-only group). The only values that were significantly increased by ACLT + low-dose HQW compared with ACLT alone were bone mineral density and trabecular numbers.

**Conclusion:**

Our findings suggest that high-dose HQW improves weight-bearing asymmetry, decreases bone loss, and reduces levels of TNF-*α* and IL-1*β* in the affected joint in ACLT-induced KOA rats. More evidence is needed to support our findings.

## 1. Introduction

Knee osteoarthritis (KOA) is a chronic, low-grade inflammatory disorder [1] that is classified as the most common joint disease worldwide, affecting around 23% of people aged 40 years and over globally [2]. The most prominent risk factors are age, obesity, female gender, and genetic factors [1]. Overuse, trauma, and malalignment are the predominant risk factors at the joint level [1]. The most commonly reported symptoms of KOA are chronic pain, joint deformity, and physical disability [3]. As many as 80% of patients with KOA are restricted in movement, 20% cannot perform basic daily activities, and 11% need personal care [4]. Thus, KOA is associated with huge societal and health care burdens, which will increase with ever-increasing numbers of KOA patients as the global population ages [1,4,5]. For instance, the number of people with KOA worldwide increased by around 17 million between 1990 and 2010 [3]. Treatment for KOA consists of surgical and nonsurgical options. In 2019, the Osteoarthritis Research Society International (OARSI) organization issued guidelines on nonsurgical strategies for patients that include exercise (land-based and water-based), weight control, strength training (e.g., quadriceps, hamstrings, and adductors), self-education, topical nonsteroidal anti-inflammatory drugs (NSAIDs), intra-articular corticosteroids (IACs), and duloxetine [6]. The OARSI guidelines strongly recommend topical NSAIDs (Level 1A) for KOA, whereas the evidence is considered to be Level 1B in regard to oral NSAIDs, IACs, COX-2 inhibitors, intra-articular hyaluronic acid (IAHA), gait aids, aquatic exercise, and self-management programs [6]. However, despite the widely accepted use of NSAIDs in the treatment of KOA, serious gastrointestinal and cardiovascular side effects can occur with these pharmacotherapies [7]. Moreover, the American College of Rheumatology (ACR)/Arthritis Foundation guidelines for the management of OA in the hand, hip, and knee conditionally recommend against IAHA and strongly recommend against glucosamine and platelet-rich plasma in patients with KOA [8]. Clearly, patients with KOA need other kinds of pharmacotherapies that are not associated with unwanted side effects.

Traditional Chinese medicine (TCM) has been utilized by countless individuals over almost 3,000 years [9]. The earliest records of TCM therapies for KOA have been found in *The Inner Classic of the Yellow Emperor* (*Huang-Di Nei-Jing*), which is believed to have been compiled during the Han dynasty (206 BCE–220 CE). It contains descriptions of herbal medicine and herbal patches, acupuncture, moxibustion, Tai-Chi, and Tui-na (manual massage) that were deemed appropriate for KOA, recorded in the text as “Gu-Bi (骨痹)” [10]. Acupuncture is strongly recommended, and Tai-Chi conditionally recommended by the ACR 2019 guidelines [6]. Clinical evidence supports the use of moxibustion for reducing intensities of OA-related pain and stiffness [11], while TCM herbal preparations have reportedly reduced inflammation and improved physical function by greater amounts, with more effective analgesia and greater safety in comparison with standard pharmacotherapies in KOA clinical trials [12]. Nonetheless, the low quality of the methodology in clinical trials using TCM herbal preparations and other TCM treatments lowers the quality of the evidence [10,12,13]. Another limitation of clinical trials using TCM preparations is that the formulas, dosages, and treatment regimens are not standardized [12]. TCM herbal preparations therefore need to be applied with more rigorous methodology to support the evidence.

The Hu-Qian-Wan (HQW) formula was first described in a book about TCM and internal medicine, *Danxi Xinfa*, compiled by Zhu Danxi in 1347 CE. HQW is used to treat people diagnosed with Yin-deficiency-heat and weakness of limbs [14]. Elderly KOA patients usually have deficient liver-Yin and kidney-Yin, accompanied by symptoms of pain, a restricted range of motion, and weakness of muscles around the knee joint. In such patients, HQW treatment has been suggested superior over other herbal formulas, due to the benefit in Yin-deficiency. The HQW formula has been used for 600 years without any record of significant adverse events. In Taiwan, the HQW formula (without the bone of Panthera tigris Linnaeus) has been used for 50 years, and no significant adverse events have been recorded since the establishment of the National Adverse Drug Reactions Reporting System in 2001. A modified formulation of HQW (substituting *Eucommia ulmoides* Oliver. for *Os Panthera tigris* Linnaeus) has been tested in patients with liver-Yin and kidney-Yin deficiency KOA and found to improve visual analogue scale (VAS) and Western Ontario and McMaster Universities Arthritis Index (WOMAC) scores by significantly greater amounts compared with diclofenac sodium over 8 weeks of treatment [15]. Nevertheless, that study was marked by methodological problems and lacks any discussion about the pharmacological mechanisms underlying the effects of modified HQW in KOA [15]. Furthermore, no animal model was included for providing any proof of the underlying mechanisms and treatment efficacy [15]. Our study sought to determine the effects of HQW in rats with KOA induced by anterior cruciate ligament transection (ACLT).

## 2. Materials and Methods

### 2.1. Materials

HQW was obtained from the Chinese Medicine Pharmacy in the Department of Chinese Medicine of China Medical University Hospital (CMUH), Taichung, Taiwan. The preparation comprised *Plastrum Testudinis*, *Phellodendron amurense* Rupr., *Anemarrhena asphodeloides* Bge., *Rehmannia glutinosa* Libosch., *Paeonia lactiflora* Pall., *Cynomorium songaricum* Rupr., *Citrus reticulata* Blanco, *Zingiber officinale* Rosc., and bone of *Capra* spp. The use of *Os Panthera tigris* Linnaeus (specified in the original formula) is forbidden by Taiwan's Animal Protection Act. All drugs were crushed into powder for study use. The COX-2-specific inhibitor celecoxib was purchased from Sigma-Aldrich (St. Louis, MO, USA). Celecoxib was selected for use in this study due to this agent having fewer gastrointestinal complications and providing longer-term pain relief compared with other NSAIDs [16]. In Taiwan, celecoxib is covered by the National Health Insurance payment system for the treatment of KOA patients with gastrointestinal comorbidities.

### 2.2. OA Animal Model

Thirty male Sprague-Dawley rats (8 weeks old; 300–350 g) were purchased from the National Laboratory Animal Center in Taipei and maintained under conditions that satisfied the guidelines issued by the Animal Care Committee of China Medical University, Taichung, Taiwan. Study approval was granted by the Animal Research Ethics Committee of China Medical University (Approval No. 2018-102). The rats were given free access to animal feed and water in a homeostatic space, according to guidelines of the Animal Care Committee of China Medical University.

OA was established by ACLT of the right knee under anesthesia with Zoletil 50® (125:125 mg of tiletamine hydrochloride and zolazepam hydrochloride), as previously detailed [17]. After the knee joint cavity was opened, the ACL was cut with micro-scissors using surgical loupes. The anterior drawer test was performed to check the success of ACLT; the joint space was cleaned with sterile saline; and then the wound was closed by suturing the capsule and skin. The rats in the control group underwent arthrotomy without transection of the ACL [18,19]. Postsurgery, the rats were fed with low-dose HQW (1,000 mg/kg), high-dose HQW (3,000 mg/kg), or celecoxib (30 mg/kg) for 6 weeks. The five study groups consisted of the sham-operated group (controls), ACLT only, ACLT + low-dose HQW, ACLT + high-dose HQW, and ACLT with celecoxib.

### 2.3. Behavioral Testing

The static weight-bearing incapacitance test (Bioseb, Paris, France) was performed every week to evaluate spontaneous pain after ACLT. This apparatus measures the difference in dynamic weight bearing between the left and right hind limbs as they rest on two separate sensor plates. We recorded forces (expressed as grams) exerted on the plates by each hind paw for 10 s after placing the rats into the apparatus. Three consecutive recordings were performed on every test day to obtain the mean score. Results are expressed as a percentage of body weight, to avoid any interference from differences in weights between animals [20, 21].

### 2.4. Micro-CT Analysis

After 6 weeks of medication, all rats were sacrificed by CO_2_ on day 49, and the right lower extremity was surgically removed from each rat. After removing the skin and muscle tissue, the intact knee joint was fixed with 3.7% formaldehyde at room temperature [19]. The right knee joints were scanned using a SkyScan 2211 high-resolution micro-computed tomography (micro-CT) scanner (Bruker, Kontich, Belgium) at the resolution of 8.5 *μ*m in saline. Micro-CT was performed using cameras that scanned over 180° of rotation with a voltage of 90 kVp, a current of 450 *μ*A (8 watt output), and a 0.5 mm aluminum filter to prevent beam hardening artifacts. Image reconstruction was performed using InstaRecon® software (Bruker, Kontich, Belgium), which also performed appropriate ring artifact and beam hardening corrections. The average gray level intensity of the reconstructed image was measured in both scans and a linear calibration was calculated between the gray-level intensity and bone mineral density (BMD). In brief, reconstructed cross-sections were reorientated, and 59 slices (0.5 mm) were selected. Images were analysed for thresholding, the regions of interest, bone morphometric analysis and BMD, bone mineral content (BMC), bone surface/total volume (BS/TV), trabecular thickness (Tb.Th), trabecular number (Tb.N), and trabecular separation (Tb.Sp) data by Bruker micro-CT software (CTAn, version 1.20.8, Bruker, Kontich, Belgium) [19, 22, 23].

### 2.5. Histological Analysis

After undergoing micro-CT scanning, the right knee joint was stored for histological examination. The tissue was fixed with 4% paraformaldehyde in phosphate-buffered saline (PBS) and kept at 37°C for 24 h and then decalcified in 10% EDTA at 4°C for 2 weeks. The decalcified tissue was dehydrated in a series of reagent ethanol solutions ranging from 70% to 100% ethanol in water and then embedded into paraffin blocks. Slices taken every 5 *μ*m of the right knee joint from each rat were stained with hematoxylin and eosin (H&E) and Safranin-O to evaluate histopathological changes under an optical microscope [24]. The OARSI histopathology grading system was used to evaluate changes in structural cartilage from the medial tibial plateau (the weight-bearing area) [25, 26], defining the grade of damage from 0 to 6 as the depth of OA progression into the cartilage and the stage of damage as the horizontal extent of cartilage damage from 0 to 4. The final score combines the grade and stage (score range 0–24). Observer bias was avoided by having two independent assessors perform the scoring of changes in the knee joint, as in previous studies [26,27].

### 2.6. Immunohistochemical Analysis

Tissue from the right knee joint was placed on slides and rehydrated. Endogenous peroxidase activity was blocked by incubating the slides in 3% hydrogen peroxide. After trypsinization, the slides were blocked again by incubation with 3% bovine serum albumin in PBS. The tissue sections were then incubated with interleukin 1 beta (IL-1*β*) or tumor necrosis factor alpha (TNF-*α*; 1:200) primary antibody at 4°C overnight, followed by incubation with secondary antibody (1:200) for 1 h at room temperature. Finally, the sections were stained with diaminobenzidine. The light microscope was used to observe the slides [24, 28]. Immunohistochemical (IHC) staining was scored from 1 to 5 (from weak to strong) for positive expression by two independent observers who were blinded to the treatment groups.

### 2.7. Statistical Analysis

Statistical calculations were performed using PRISM 5.0 software (GraphPad, San Diego, CA, USA). Between-group differences were assessed for significance using the unpaired two-tailed Student's *t*-test and analysis of variance (ANOVA). All results are expressed as the mean ± standard error of the mean (S.E.M.). A *p* value of less than 0.05 was considered to be statistically significant.

## 3. Results

### 3.1. High-Dose HQW Improved Weight-Bearing Asymmetry

**Figure 1:**
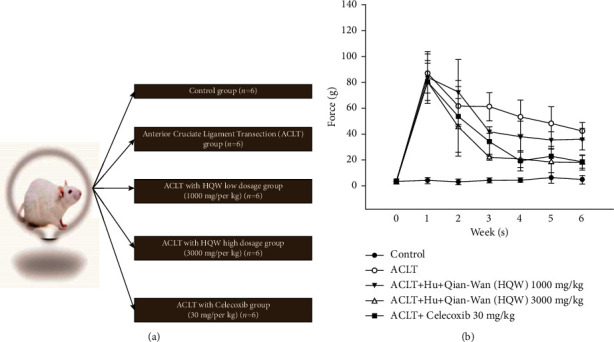
(a) The study groups after ACLT surgery in Sprague Dawley rats. (b) Development of weight-bearing deficits in rat hind legs after sham surgery (controls), ACLT alone, ACLT in combination with low-dose HQW (1,000 mg/kg) or high-dose HQW (3,000 mg/kg), or ACLT in combination with celecoxib (30 mg/kg) and the effects of the different interventions on those deficits at 6 weeks.

After surgery, the effects of low- and high-dose HQW, as well as celecoxib, were compared with no such treatment on asymmetric weight-bearing posture in sham-operated controls and ACLT-only rats (Figure 1(a)). After 6 weeks, the smallest differences in weight-bearing asymmetry between the postoperative and normal hind limbs were observed with high-dose HQW (17.80 ± 5.68 g) and celecoxib (18.68 ± 5.29 g) compared with ACLT only (42.17 ± 6.68 g) and low-dose HQW (36.00 ± 8.25 g; Figure 1(b)). By week 3, high-dose HQW was associated with the least amount of asymmetry when compared with all other study groups (Figure 1(b)).

### 3.2. High-Dose HQW Improved Bone Architecture

**Figure 2:**
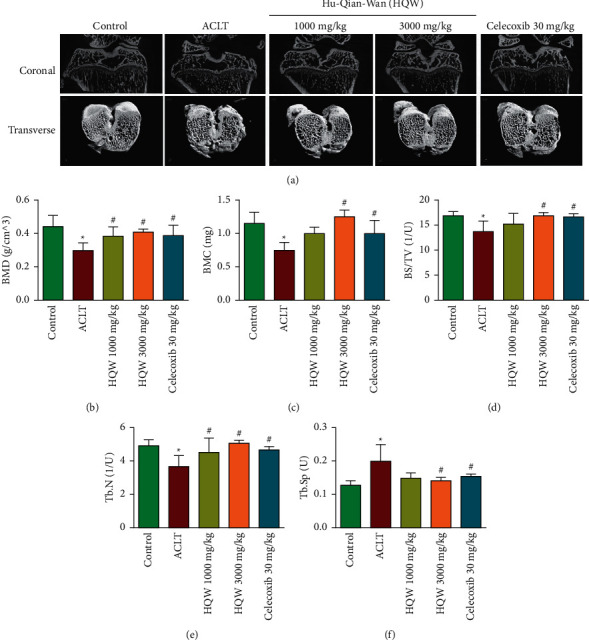
(a) Representative micro-CT images of the right knee joint from rats in the control group, the ACLT-only group, the ACLT + low-dose HQW group, the ACLT + high-dose HQW group, and the ACLT + celecoxib group. The upper panel depicts coronal and the lower panel depicts transverse (3D visualization) images of proximal tibias from all study groups (six rats/group). (b) Bone mineral density (BMD). (c) Bone mineral content (BMC). (d) Bone surface/total volume (BS/TV). (e) Trabecular bone number (Tb.N). (f) Trabecular bone space (Tb.Sp). ^*∗*^*p* < 0.05 versus the control group; ^#^*p* < 0.05 versus the ACLT-only group.

Figure 2(a) depicts apparently superior Tb.Th and Tb.N micro-CT measurements with high-dose HQW and celecoxib compared with ACLT alone. As shown in Figures 2(b)–2(f), micro-CT measurements differed significantly between controls and ACLT-only rats for BMD, BMC, BS/TV, Tb.N, and Tb.Sp, indicating efficient establishment of the KOA animal model (*p* < 0.05 for all comparisons). BMD, BMC, BS/TV, Tb.N, and Tb.Sp values were all significantly superior with ACLT + high-dose HQW and ACLT + celecoxib compared with ACLT alone (*p* < 0.05 for all comparisons), while BMD, BMC, and Tb.N values were slightly better with ACLT + high-dose HQW compared with ACLT + celecoxib. BMD and Tb.N values were significantly superior with ACLT + low-dose HQW compared with ACLT alone (*p* < 0.05 for all comparisons).

### 3.3. High-Dose HQW Protected Articular Cartilage against ACLT-Induced Damage

**Figure 3:**
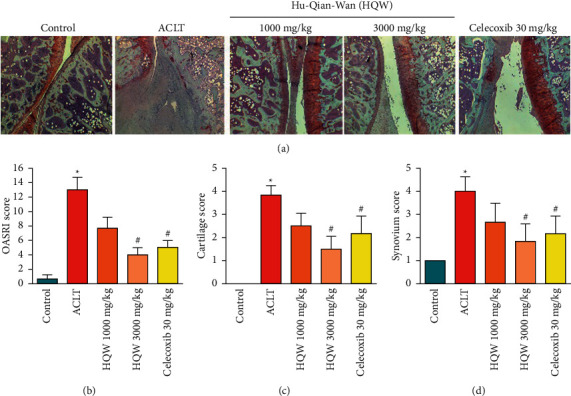
Histological analysis of structural damage in articular cartilage. (a) Coronal sections of articular cartilage from the knee joint in the different groups stained with Safranin-O (magnification 5x). (b) OARSI total scores were calculated based on histological staining. (c) OARSI cartilage scores. (d) OARSI scoring of synovial inflammation. ^*∗*^*p* < 0.05 versus the control group; ^#^*p* < 0.05 versus the ACLT-only group.

Figure 3(a) shows more intact Safranin-O staining (indicating less injury) in articular cartilage samples in the ACLT + low-dose HQW group, ACLT + high-dose HQW group, and the ACLT + celecoxib group compared with the ACLT-only group, in which the cartilage is almost invisible. OARSI, cartilage, and synovial scores (Figures 3(b)–3(d)) were significantly superior in the ACLT + high-dose HQW group and the ACLT + celecoxib group compared with scores in the ACLT-only group (*p* < 0.05 for all comparisons).

### 3.4. High-Dose HQW Downregulated Levels of TNF-*α* and IL-1*β* in the Cartilage and Synovium

**Figure 4:**
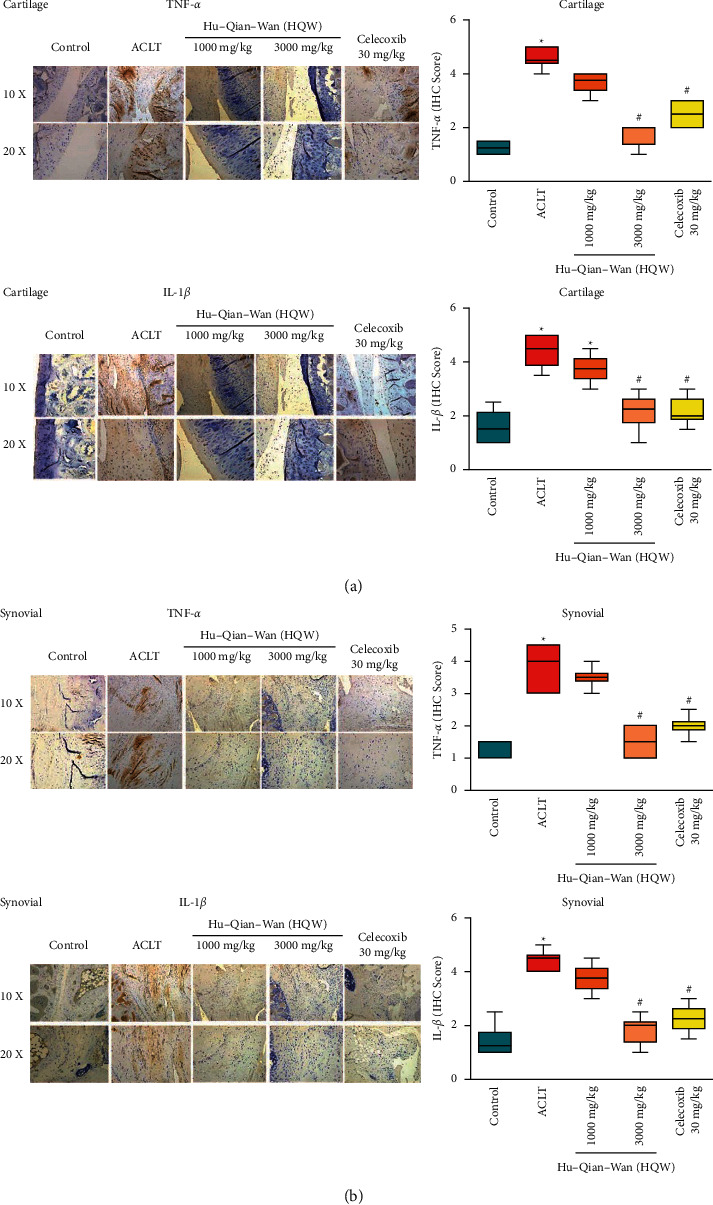
Histological analysis of structural damage in articular cartilage. (a) Coronal sections of articular cartilage from rats in each group were stained with TNF-*α* and IL-1*β* antibodies. IHC scores for TNF-*α* and IL-1*β* levels of expression in articular cartilage. (b) IHC staining for TNF-*α* and IL-1*β* antibodies in synovium sections from all rats. IHC scores for TNF-*α* and IL-1*β* levels of expression in synovial tissue. ^*∗*^*p* < 0.05 versus the control group; ^#^*p* < 0.05 versus the ACLT-only group.

As shown in Figure 4(a), TNF-*α* and IL-1*β* expression was upregulated by the greatest amount in cartilage chondrocytes from the ACLT-only group compared with all other study groups, while ACLT + high-dose HQW was associated with the lowest amounts of TNF-*α* and IL-1*β* expression. The numbers of TNF-*α* and IL-1*β* immunoreactive cells were significantly lower with ACLT + high-dose HQW and ACLT + celecoxib compared with the ACLT-only group. TNF-*α* expression was slightly lower with ACLT + high-dose HQW compared with celecoxib.

Figure 4(b) depicts the highest amounts of TNF-*α* and IL-1*β* expression in synovium from the ACLT-only group compared with all other study groups, while ACLT + high-dose HQW was associated with the lowest amount of TNF-*α* and IL-1*β* staining in synovium. The between-group differences were significant for TNF-*α* and IL-1*β* expression with both ACLT + high-dose HQW and ACLT + celecoxib compared with ACLT alone. Levels of TNF-*α* and IL-1*β* expression were slightly lower with ACLT + high-dose HQW than with ACLT + celecoxib.

## 4. Discussion

Degeneration of the cartilage and synovium in the joints increases functional disability in elderly patients with KOA [1, 27]. In our experimental rat model of KOA, HQW improved weight-bearing distribution, protected the cartilage and synovium from injury by decreasing inflammation in the knee joint, and reduced the loss of bone content. The HQW formula includes *Chinemys reevesii* (Gray), *P. amurense* Rupr., *A. asphodeloides* Bunge, *R. glutinosa* Libosch., *P. lactiflora* Pall., *C. songaricum* Rupr., *C. reticulata* Blanco, *Z. officinale* Roscoe, and bone of *Capra* spp. Cellular and preclinical evidence has demonstrated that these herbs have anti-inflammatory properties and reduce oxidative stress, inhibit apoptosis, provide analgesia, prevent cartilage degradation by downregulating levels of matrix metalloproteinases (MMPs) and “aggrecanase,” a disintegrin and metalloproteinase with thrombospondin motifs (ADAMTSs), and regulate the proliferation of rat marrow-derived mesenchymal stem cells (rMSCs) [29,30]. For example, ethyl acetate extraction of Ts-12 from *P. Testudinis* induces the proliferation of rMSCs [31]. *P. amurense* (PA) can inhibit IL-1*α*-induced degradation of glycosaminoglycan (GAG) and type II collagen from human osteoarticular cartilage [32]. PA can also decrease the expression of aggrecanase-1, aggrecanase-2, MMP-1, MMP-3, MMP-13, the phosphorylation of c-Jun NH2-terminal kinase (JNK), p38 mitogen-activated protein kinase (MAPK), and extracellular signal-regulated kinase (ERK) 1/2 during OA changes in chondrocytes [32]. Moreover, PA can increase levels of TIMP-1, an inhibitory molecule that regulates MMP expression [32]. In an 8 week randomized, double-blind study, patients with OA were treated with extracts of *P. amurense* bark and *Citrus sinensis* peel or placebo [33]. At 8 weeks, Lequesne Algofunctional Index (LAI) scores were significantly superior in the TCM treatment group compared with those in the placebo group [33]. Extracts of Timosaponin B-II from *A. asphodeloides* Bge. reduce nuclear factor kappa B (NF-*κ*B) mRNA and protein levels and regulate MAPK signaling in lipopolysaccharide-stimulated BV2 cells [34]. The extracts of *R. glutinosa* Libosch affect the activity of osteoblasts and osteoclasts by increasing the proliferation and alkaline phosphate activity of osteoblasts, decreasing the loss of trabecular bone, and increasing the thickness of cortical bone in ovariectomy-induced osteoporotic rats [35]. Rehmanniae Radix Preparata from *R. glutinosa* Libosch. can prevent osteoporosis (in postmenopausal and elderly patients) by inhibiting glucocorticoid-induced osteoporosis and improving BMD, especially in trabecular bone [36]. Total glycosides of paeony (TGP) extracted from *P. lactiflora* Pall. exhibits anti-inflammatory properties and regulates immune cells and various signaling pathways (e.g., the G protein-coupled receptor (GPCR) pathway, the MAPK/NF-*κ*B pathway, the PI3K/Akt/mTOR pathway, and transforming growth factor beta (TGF-*β*)/Smad signaling, amongst others) in autoimmune diseases [37]. Monoterpene glycosides extracted from *P. lactiflora* Pall. have wide-ranging effects, including anti-inflammatory and antioxidative properties, analgesic effects, immunoregulatory and cardioprotective activities, as well as neuroprotective and hepatoprotective effects [38]. TGP can also inhibit joint destruction in rats with collagen-induced arthritis by decreasing the secretion or production of vascular endothelial growth factor (VEGF), basic fibroblast growth factor (bFGF), MMP-1, and MMP-3 from fibroblast-like synoviocytes [39]. Extract of *C. songaricum* Rupr. displays neuroprotective effects in ovariectomized rats, possibly by p-cAMP-response element binding protein (p-CREB) and brain-derived neurotrophic factor (BDNF) mediating the downregulation of ERK/p38MAPK levels [40]. Phenolic antioxidants epicatechin and luteolin-7-O-*β*-D-glucoside extracted from *C. songaricum* Rupr. exhibit superior antioxidative activities over those of other phenolic antioxidants in •OH-damaged MSCs [41]. The 39 flavonoid compounds identified in citrus (*Citrus reticulate* Blanco) fruit, particularly the flavonoid glycosides didymin, hesperidin, neohesperidin, poncirin, and naringin, display marked anti-oxidative activity [42]. Ginger root extract has demonstrated antiarthritic effects in sow OA cartilage explants, reducing nitric oxide and prostaglandin E2 production [43]. Whereas investigations using celecoxib in human OA cartilage have found that this compound fails to significantly inhibit the release of GAG induced by IL-1*α*, celecoxib can effectively suppress MMP-1 and MMP-13 levels [32]. The interactions of the herbs in the HQW formula may induce therapeutic effects in KOA through these various mechanisms mentioned above.

The findings of this study suggest that decreases in levels of TNF-*α* and IL-1*β* expression may be due to berberine content in Phelloendri Cortex. Paeoniae Alba Radix and Anemarrhenae Rhizoma can lower TNF-*α* and IL-1*β* expression by regulating the NF-*κ*B pathway. The effect of cartilage protection may be due to Paeoniae Alba Radix and Phelloendri Cortex, which are capable of decreasing MMP-1 and MMP-3 and are responsible for inducing cartilage degradation. Improvements in weight-bearing distribution may be due to a reduction in pain associated with the anti-oxidant and anti-inflammatory activities of Phelloendri Cortex, Anemarrhenae Rhizoma, Paeoniae Alba Radix, Cynomorii Herba, Citri Reticulatae Pericarpium, and Zingiberis Rhizoma. Improvements in BMD may be due to Rehmanniae Radix Praeparata, which increases osteoblast activity and inhibits glucocorticoid-induced osteoporosis. All of these speculations need more evidence for proof of concept.

There were no significant differences in this study between celecoxib and high-dose HQW for their effects on weight-bearing distribution, protection of cartilage, amount of bone loss, and knee joint levels of TNF-*α* and IL-1*β*. Notably, whereas celecoxib is associated with an increased risk of death due to cardiovascular causes [44], yet the use of the HQW formula has not been reported with related adverse event over the last 20 years in Taiwan. Thus, HQW may represent a valid therapeutic alternative to celecoxib for treating patients with KOA.

Three limitations must be mentioned in regard to this study. The first major limitation is the small sample size. Second, the major mechanisms remain undefined, and further study is therefore needed to determine these possible mechanisms that support the effects of HQW. Third, it remains necessary to isolate and purify the bioactive compounds in the HQW formula.

Taiwan's Ministry of Health and Welfare has granted approval for the medical use of the HQW formula. More *in vitro* and preclinical investigations are needed to examine the underlying therapeutic mechanisms of the HQW formula. Moreover, a well-designed, double-blind, placebo-controlled trial is needed to examine the therapeutic effects of HQW in patients with KOA.

## 5. Conclusions

This study has shown that the HQW formula improves weight-bearing asymmetry, protects the knee joint by downregulating IL-1*β* and TNF-*α* expression, and reduces the loss of bone in rats with ACLT-induced KOA. This study needs more evidence to support these results.

## Data Availability

The data used to support the findings of this study are available from the corresponding author upon request.

## Abbreviations

ACL: Anterior cruciate ligament
ACLT: Anterior cruciate ligament transection
ACR: American college of rheumatology
ADAMTSs: A disintegrin and metalloproteinase with thrombospondin motifs
BDNF: Brain-derived neurotrophic factor
bFGF: Basic fibroblast growth factor
BMC: Bone mineral content
BMD: Bone mineral density
BS/TV: Bone surface/total volume
CMUH: China medical university hospital
COX-2: Cyclooxygenase-2
EDTA: Ethylenediaminetetraacetic acid
ERK: Extracellular signal-regulated kinase
GAG: Glycosaminoglycan
GPCR: G protein-coupled receptor
H&E: Hematoxylin and eosin
HQW: Hu-Qian-Wan
IACs: Intra-articular corticosteroids
IAHA: Intra-articular hyaluronic acid
IHC staining: Immunohistochemical staining
IL-1*β*: Interleukin 1 beta
JNK: c-Jun NH2-terminal kinase
KOA: Knee osteoarthritis
LAI score: Lequesne algofunctional index score
MAPK: Mitogen-activated protein kinase
micro-CT: Micro-computed tomography
MMPs: Matrix metalloproteinases
NF-*κ*B: Nuclear factor kappa B
NSAIDs: Nonsteroidal anti-inflammatory drugs
OARSI: Osteoarthritis research society international organization
PA: *Phellodendron amurense*
PBS: Phosphate-buffered saline
p-CREB: p-cAMP-response element binding protein
rMSCs: Rat marrow-derived mesenchymal stem cells
Tb.N: Trabecular number
Tb.Sp: Trabecular separation
Tb.Th: Trabecular thickness
TCM: Traditional Chinese medicine
TGF-*β*: Transforming growth factor beta
TGP: Total glycosides of paeony
TNF-*α*: Tumor necrosis factor alpha
VAS: Visual analogue scale
VEGF: Vascular endothelial growth factor
WOMAC: Western Ontario and mcmaster universities arthritis index.
